# Pyridin-3-carbohydrazide strongly impairs Mayaro and Chikungunya virus replication *in vitro*

**DOI:** 10.1007/s00705-026-06709-y

**Published:** 2026-08-12

**Authors:** Nancy Araceli Juárez Contreras, Natasha Marques Cassani, Mikaela dos Santos Marinho, Giulia Magalhães Ferreira, Maria Eduarda Trevisan Aguillar da Silva, João Victor Rodrigues Dutra, Juliana Roberta Torini, Sainan Wang, Kelyssa Vitória Rodrigues Dantas, Isabela Dolci, Mariana Ortiz de Godoy, Lavinia Cipriano, Margus Varjak, Rafaela Sachetto Fernandes, Rafael Victorio Carvalho Guido, Glaucius Oliva, Bo Zhang, Andres Merits, Bruno Amaral Meireles, Diego Pandeló José, Ana Carolina Gomes Jardim

**Affiliations:** 1 Laboratory of Antiviral Research, Institute of Biomedical Science (ICBIM), Federal University of Uberlândia (UFU), Av. Amazonas, 4C Building – room 216, Uberlândia, Minas Gerais 38405-302 Brazil; 2 Federal University of Triângulo Mineiro, Campus Universitário Iturama, Iturama, Minas Gerais Brazil; 3 Laboratory of Integrative Biology, Institute of Biological Sciences, Federal University of Minas Gerais (UFMG), Belo Horizonte, Minas Gerais Brazil; 4 Center for Research and Innovation in Biodiversity and Pharmaceuticals, São Carlos Institute of Physics (IFSC), University of São Paulo (USP), São Carlos, São Paulo, Brazil; 5 Institute of Bioengineering, University of Tartu, Tartu, Estonia; 6 Key Laboratory of Special Pathogens and Biosafety, Center for Biosafety Mega-Science, Wuhan Institute of Virology, Chinese Academy of Sciences, Wuhan, China; 7 Institute of Biosciences, Humanities and Exact Science (IBILCE), São Paulo State University (UNESP), São José do Rio Preto, São Paulo, Brazil

## Abstract

Alphaviruses, including Mayaro virus (MAYV) and Chikungunya virus (CHIKV), pose a growing public health threat, with increasing reports worldwide and no licensed antiviral treatments available. This study evaluated the antiviral activity of pyridine-3-carbohydrazide (P-3-C) against MAYV (strains BeAr and TRVL) and CHIKV (strain La Reunion, LR). Vero E6 or U-2OS cells were infected and treated with P-3-C for 24 h, and cell viability and replication levels were assessed. The 50% cytotoxic concentration (CC_50_), 50% effective concentration (EC_50_), and selective index (SI) were calculated. P-3-C showed concentration-dependent activity, strongly inhibiting post-entry stages of MAYV BeAr replication. Treatment reduced intracellular viral RNA levels by 2 log_10_ compared with controls and effectively decreased viral RNA release. P-3-C also inhibited MAYV TRVL (SI > 29.4) and CHIKV LR (SI > 48.8) in Vero E6 cells and retained selectivity in U-2OS cells (SI = 3.9 and 6.4, respectively). In a CHIKV U-2OS subgenomic replicon, an SI of 4.1 was found. Additionally, Fluorometric FRET-Based and MicroScale Thermophoresis (MST) assays indicated that P-3-C inhibited the nsP2 protease activity (IC_50_ = 12 µM ± 2) and exhibited binding affinity for nsP4 polymerase (*K*_d_ = 54 µM ± 1) from CHIKV, thereby suggesting that these non-structural proteins are potential molecular targets. Molecular docking positioned P-3-C within the catalytic pockets of nsP2 and nsP4 of both viruses. Overall, the data suggest that P-3-C exhibits a potential multitarget mechanism of action and highlight its antiviral activity against different alphavirus strains; however, this efficacy could be dependent on the cell line used.

**Supplementary Information:**

The online version contains supplementary material available at https://doi.org/10.1007/s00705-026-06709-y.

## Introduction

Alphaviruses such as Mayaro virus (*Alphavirus mayaro* - MAYV) and chikungunya virus (*Alphavirus chikungunya* - CHIKV) (*Togaviridae* family) are transmitted by the bite of mosquitoes [1]. After infecting the host, the virus spreads through the bloodstream, causing a wide range of symptoms, including diarrhea, muscle aches, and retroorbital pain, as well as local inflammatory processes [2]. Inflammation can lead to arthralgia and arthritis that persist in 54% of cases, even after 12 months [2, 3]. Mayaro fever has been reported mainly in Central and South America; however, imported cases have been documented in North America and Europe [1, 4]. Chikungunya fever has been reported in America, Africa, and Asia [5].

MAYV and CHIKV have enveloped virions with diameters of 65–70 nm and positive-strand RNA genomes of about 11.5 kb, with two open-reading frames (ORFs). ORF1, located at the 5´end of the genome, encodes the precursor of non-structural proteins (nsP1, nsP2, nsP3, and nsP4) involved in viral replication [6]. ORF2, located at the 3´end of the genome, encodes precursors of structural proteins (capsid protein, E3, E2, 6 K, and E1), which are essential for the virion formation [6].

As of now, the FDA has approved a live-attenuated vaccine to prevent CHIKV infection. The lack of vaccines against other alphaviruses or approved antiviral treatments against any of them highlights the urgent need to search for new therapeutic options [7]. In this context, natural and synthetic compounds have been investigated as possible candidates for antiviral drug development [8–12].

Pyridin-3-carbohydrazide (P-3-C), also known as nicotinic acid hydrazide, niazid, 3-pyridoyl hydrazine, or nicotinohydrazide [13], is a heterocyclic compound with a molecular formula of C_6_H_7_N_3_O. It consists of a pyridine ring attached to a carbohydrazide group (-CONHNH_2_) at the 3-position [14]. P-3-C has attracted considerable interest due to its wide range of biological activities, including antitumoral, antimicrobial, antifungal, antituberculosis, and anti-inflammatory properties [15–19]. In this context, this study assessed the in vitro and in silico antiviral effects of P-3-C on the replicative cycle of MAYV and CHIKV.

## Materials and methods

### Cell culture

Vero E6 cells (African green monkey kidney cells; ATCC CRL-1586) and U-2OS cells (human osteosarcoma cells; ATCC.HBT-96) were cultured in Dulbecco’s Modified Eagle’s Medium (DMEM, Sigma-Aldrich, Missouri, USA) or Iscove’s Modified Dulbecco’s Medium (IMDM, Corning, Massachusetts, USA), respectively, in a humidified 5% CO_2_ incubator at 37 °C. DMEM and IMDM were supplemented with 100 mg/mL penicillin (Gibco Life Technologies, Massachusetts, USA), 100 mg/mL streptomycin (Gibco Life Technologies, Massachusetts, USA), and 1% (v/v) of stock of non-essential amino acids (Gibco Life Technologies, Massachusetts, USA) and 5% (v/v) of fetal bovine serum (FBS) (Cultilab, Brazil).

### Plasmids, viruses, and compound

**Fig. 1:**
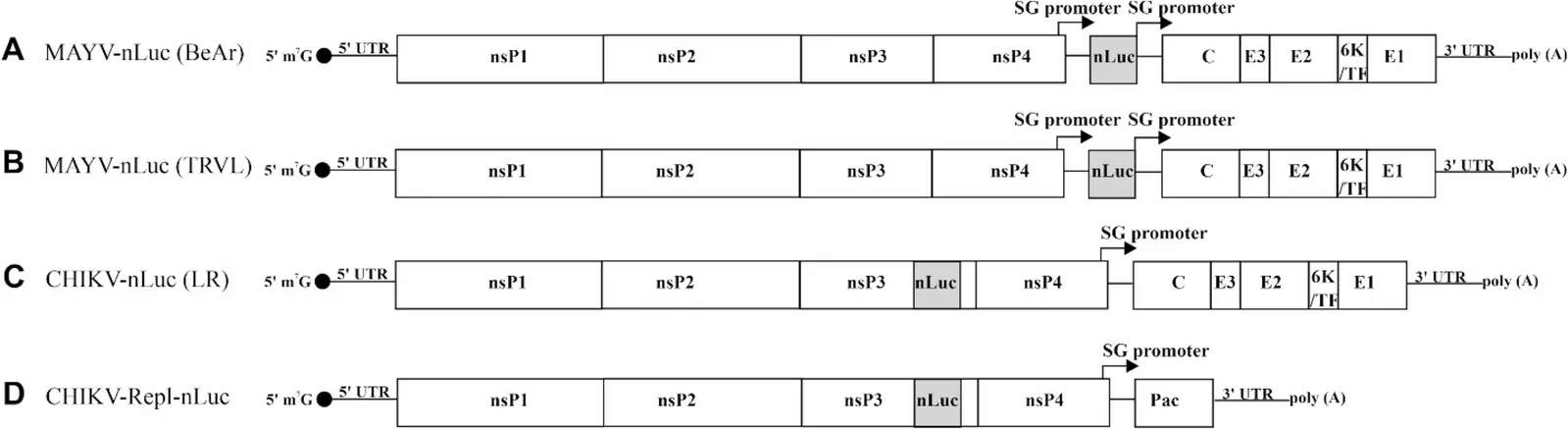
Schematic representation of plasmids used in this study. **A** MAYV-nLuc-BeAr; **B** MAYV-nLuc-TRVL; **C** CHIKV-nLuc-LR; **D** CHIKV-Repl-nLuc subgenomic replicon

The plasmid containing the infectious cDNA (icDNA) for MAYV BeAr 20290 strain (genotype L) [20], with the sequence of the nanoluciferase (nLuc) reporter inserted between ORF1 and ORF2 (Fig. 1A) [21], was used to produce MAYV-nLuc BeAr virions. Alternatively, a novel plasmid based on the MAYV TRVL strain (genotype D) was developed using a previously established icDNA clone of MAYV TRVL [22] as a backbone, with modifications that included replacing the SP6 promoter with a CMV promoter, insertion of nLuc and a duplicated subgenomic promoter (-80/+27 relative to the start of SG RNA) downstream of ORF1, and adding a hepatitis delta virus (HDV) ribozyme and Simian virus 40 (SV40) terminator downstream of the poly-A tail at the 3´end of MAYV cDNA (Fig. 1B)**.** The final sequence of the plasmid, designated CMV-MAYV-nLuc TRVL, was confirmed by sequencing.

Additionally, a plasmid containing the icDNA of CHIKV-nLuc, which was based on the CHIKV LR2006-OPY1 strain (East/Central/South African genotype), with the nLuc inserted into the C-terminal domain of viral nsP3 protein (Fig. 1C) [23, 24], was used.

MAYV-nLuc-BeAr, MAYV-nLuc-TRVL, and CHIKV-nLuc were rescued as previously described [21, 25].

Pyridine-3-carbohydrazide (P-3-C) was purchased from Sigma-Aldrich (CAS:553-53-7; cat. No. 107425), dissolved in dimethyl sulfoxide (DMSO) at a stock concentration of 50 mM, and stored at 20 °C. DMSO at 0.1% was used as untreated control.

The cell-culture-adapted, non-cytotoxic replicon of CHIKV LR, designated CHIKV-Repl-nLuc, harboring the nLuc reporter in the nsP3 and puromycin acetyltransferase selection marker (Pac) under the subgenomic promoter (Fig. 1D), was used.

### Viability assays

The cytotoxic of the compound was evaluated using the WST-1 (water-soluble tetrazolium salt-1) assay. Vero E6 cells or U-2OS cells were seeded in 96-well plates at a density of 2 × 10^4^ cells per well and incubated overnight at 37 °C with 5% CO_2_. Medium containing P-3-C at concentrations ranging from 1.56 to 200 µM in two-fold serial dilutions was added to the cell culture. After incubation for 24 h at 37 °C, 10 µl of WST-1 reagent (Merck, Darmstadt, Germany) was added to each well and incubated at 37 °C for 1.5 h. Absorbance was measured as the optical density (OD) at 450 nm using a GloMax microplate reader (Promega, Wisconsin, USA). Cell viability was calculated using the equation (T/C) x 100%, where T and C represent the mean OD of the treated and vehicle control groups, respectively. CC_50_ was calculated using GraphPad Prism 8.0 for Windows (GraphPad Software, Boston, Massachusetts, USA).

### Antiviral assays

To determine the 50% effective concentration (EC_50_), Vero E6 or U-2OS cells were seeded in 96-well plates at a density of 2 × 10^4^ cells per well and incubated overnight at 37 °C with 5% CO_2_. Subsequently, cells were infected with MAYV-nLuc-BeAr, MAYV-nLuc-TRVL, or CHIKV-nLuc at a multiplicity of infection (MOI) of 0.1, and P-3-C was added at concentrations ranging from 1.56 to 200 µM in two-fold serial dilutions. The lower MOI allows the ability of the compound to reduce viral infectivity to be assessed under conditions that more closely approximate a natural, multi-cycle infection [21]. Cells were then incubated for 24 h at 37 °C with 5% CO_2_. After incubation, samples were harvested using *Renilla* Luciferase Lysis Buffer (Promega, Wisconsin, USA), and luminescence levels were quantified using *Renilla* Luciferase Assay System with the GloMax microplate reader (Promega, Wisconsin, USA). Replication rates were calculated using the equation (T/C) x 100%, where T and C represent the mean luminescence of the treated and vehicle control groups, respectively. EC_50_ was calculated using GraphPad Prism 8.0 for Windows (GraphPad Software, Boston, Massachusetts, USA). The EC_50_ and CC_50_ values were used to calculate the selectivity index (SI = CC_50_/EC_50_).

### Time-of-drug-addition assay

For all time-of-drug-addition assays, Vero E6 cells were seeded in 48-well plates at a density of 5 × 10^4^ cells per well and incubated overnight at 37 °C with 5% CO_2_. Cells were treated with P-3-C at 25 µM, and virus replication rates were evaluated by measuring nLuc activity 24 h post-infection (h.p.i). Infections were performed using MAYV-nLuc-BeAr at an MOI of 1 (to synchronize replication cycle) [26], except for the virucidal assay, in which an of 5 was used (in order to challenge the compound with a higher viral load prior to cell infection) [27]. DMSO at 0.1% served as the untreated control.

In the pretreatment assay, cells were treated with P-3-C for 1 h before infection. Subsequently, cells were washed with PBS, and MAYV-nLuc-BeAr was added for 1 h. After this incubation, cells were washed with PBS to remove the unbound virus, and fresh medium was added. Cells were then incubated for 24 h at 37 °C with 5% CO_2_.

In the entry inhibition assay, cells were infected using media containing both the compound and virus for 1 h Cells were washed with PBS and incubated with fresh medium for 24 h at 37 °C with 5% CO_2_.

Virucidal activity was evaluated using the same protocol as the entry assay, with the addition of an initial step in which the virus-compound inoculum was incubated at 37 °C for 1 h before being added to the cells.

In the post-entry assay, cells were infected with MAYV-Nluc-BeAr for 1 h, washed with PBS to remove unbound particles, and then incubated with drug-containing medium for 24 h at 37 °C with 5% CO_2_. To analyze the effect of the compound on the virion release, the post-entry protocol was followed; however, supernatant and adherent cells were separately harvested 24 h.p.i. for virus quantification by reverse transcription quantitative PCR (RT-qPCR).

### Virus RNA quantification by RT-qPCR

RNA was extracted from supernatant containing virus and adherent cells using an RNA extraction kit (QIAGEN, Hilden, Germany), according to the manufacturer’s instructions. The extracted RNA was used for cDNA synthesis using the High-Capacity cDNA Reverse Transcription Kit (Applied Biosystems, Massachusetts, USA). The RT-qPCR was performed using ReadyMix JumpStart^™^ Taq SYBR^®^ Green^™^ (Sigma-Aldrich, Darmstadt, Germany) to detect the amplification of alphaviruses’ nsP1 region (forward primer: YAGAGCDTTTTCGCAYSTRGCHW; reverse primer: ACATRAANKGNGTNGTRTCRAANCCDAYCC), as previously described [28]. Reactions were run on the Applied Biosystems 7500 Fast Real-Time PCR System (Branchburg, NJ, USA). Absolute quantification was performed by interpolation from a standard curve constructed with ten-fold serial dilutions of RNA ranging from 10^1^ to 10^7^ copies per reaction. The curve showed an amplification efficiency of 100%. The assay limit of detection (LOD), defined as the lowest concentration consistently detected, was 100 copies per reaction. Samples showing no amplification by cycle 35 were considered below the assay detection limit.

### Replication assay using stable U-2OS cell line harboring CHIKV-Repl-nLuc

U-2OS-CHIKVsg cells, which harbor a non-cytotoxic CHIKV-Repl-nLuc replicon, were seeded at a density of 1 × 10^4^ cells per well in 96-well plates and incubated overnight at 37 °C with 5% CO_2_. IMDM containing serial dilutions of P-3-C, at concentrations ranging from 1.56 µM to 200 µM, was added to cells and incubated for 48 h. Cells were then harvested using *Renilla* Luciferase Lysis Buffer (Promega, Wisconsin, USA), and luminescence levels were quantified using *Renilla* Luciferase Assay System with the GloMax microplate reader (Promega, Wisconsin, USA). In parallel, cell viability was assessed using the WST-I assay. EC_50_ and CC_50_ were calculated using GraphPad Prism 8 software, and the SI was determined as the ratio of CC_50_ to EC_50_.

### Expression and purification of CHIKV nsP2 and nsP4

The expression, purification, and inhibition assays of CHIKV nsP2pro were performed as described by Cipriano (2025) and based on protocols by Narwal et al. (2018) [29, 30]. The pET-TRX expression clones containing the nsP2pro coding sequences were kindly provided by Dr. Michael Fairhead (University of Oxford). Briefly, the recombinant proteins were expressed in *Escherichia coli* BL21(DE3). Following cell lysis, the proteins were purified via Ni-affinity chromatography. The N-terminal 6xHis-tag was cleaved by TEV protease, and a final purification step was conducted by size-exclusion chromatography (Superdex 75) in a buffer containing 25 mM HEPES (pH 7.5), 150 mM NaCl, 5% glycerol, and 0.5 mM TCEP.

The CHIKV nsP4 cloned in pFastBac-Bse vector was expressed in Sf9 insect cells using a baculovirus expression vector (BEV) system [31]. The recombinant protein was purified by a four-step chromatographic procedure consisting of nickel-affinity chromatography, heparin-affinity chromatography, anion-exchange chromatography on a HiTrap^®^ DEAE column, and size-exclusion chromatography on a Superdex 200 column. The His-tag was subsequently removed by Tobacco Etch Virus (TEV) protease digestion, and the cleavage mixture was subjected to a second nickel-affinity chromatography step to separate the cleaved tag and TEV protease from the untagged nsP4 protein [32].

### MicroScale thermophoresis (MST)

MicroScale Thermophoresis experiments were carried out using the Monolith^®^ NT.115 instrument (Nanotemper Technologies, München, Germany), as previously described [32]. The CHIKV nsP4 was labeled on Cys residues with the fluorescent probe NT-647-Maemide dye 115 (Nanotemper Technologies, München, Germany). The protein concentration used in the thermophoresis experiments was 40 nM with 4% MST power and 40% LED power. The range of P-3-C concentrations tested was 2.5 − 7.6 × 10^− 5^ mM.

### Fluorometric FRET-based assay

Protease inhibitory activity was evaluated using a FRET-based enzymatic assay. Reactions were conducted at 30 °C in an assay buffer (25 mM HEPES pH 7.5, 0.01% Triton X-100) containing 600 nM of protease and 20 µM of the fluorogenic substrate DABCYL-DRAGG↓YIFSEE-EDANS-NH₂. Fluorescence was continuously monitored for 2 h (excitation/emission at 340/490 nm) using a SpectraMax i3 microplate reader. The percentage of inhibition was determined by comparing the initial reaction rates of the tested compounds against a negative control containing the enzyme, substrate, and 1% DMSO. The range of P-3-C concentrations tested was 500 − 0.12 µM. The half-maximal inhibitory concentration (IC_50_) values and concentration-response curves were calculated using GraphPad Prism software (version 8.4.3), applying a nonlinear regression analysis.

### Structural modeling and molecular docking

The three-dimensional structures of the non-structural proteins 2 (nsP2) and 4 (nsP4) of CHIKV and MAYV were predicted from their amino acid sequences using the AlphaFold3 algorithm, accessed via the AlphaFold Server (https://alphafoldserver.com/). The sequences were retrieved from the UniProt database using the accession IDs Q8JUX6 (CHIKV, strain S27-African prototype) and Q8QZ73 (MAYV, strain Brazil). As both entries correspond to the complete non-structural polyprotein (P1234), the mature nsP2 (798 residues) and nsP4 (611 residues) chains were extracted according to the chain annotations of each entry and used as input for modelling. To assess the extent to which the CHIKV enzymes represent their MAYV counterparts, the corresponding chains of the two viruses were aligned pairwise using the Needleman-Wunsch global alignment algorithm with the BLOSUM62 substitution matrix (Supplementary Table S1; Fig. S1).

The AlphaFold-predicted structures correspond to ligand-free (apo) conformational states of the nsP2 and nsP4 proteins and were used as starting models for subsequent analyses. While these initial models represent apo states, molecular docking simulations were conducted to evaluate the binding interactions with pyridine-3-carbohydrazide (P-3-C). The highest-confidence models were selected for subsequent analyses based on integrated validation metrics: the predicted Local Distance Difference Test (pLDDT) for local per-residue confidence, the predicted Template Modeling score (pTM) for global topology accuracy, and the Predicted Aligned Error (PAE) for the accuracy of relative domain orientations.

Molecular docking was performed with AutoDock Vina v1.1.2 [33] to investigate the interactions of pyridine-3-carbohydrazide (P-3-C) with the 3D structures of the nsP2 and nsP4 of CHIKV and MAYV. Receptor and ligand preparation were carried out using AutoDockTools v1.5.7. For the receptors, water molecules and heteroatoms were removed, followed by the addition of polar hydrogens and assignment of Kollman partial charges. Ligand preparation included the prediction of ionization states at physiological pH (7.4), assignment of Gasteiger charges, merging of non-polar hydrogens, and definition of torsional degrees of freedom (TORSDOF). For the nsP2 protease, the search space was centered on the catalytic residue Cys478. For the nsP4 RdRp, the search space was centered on the catalytic residue Asp466. For all targets, grid boxes were defined with dimensions of 20 Å × 20 Å × 20 Å. Docking calculations were performed using an exhaustiveness value of 24, generating up to 20 binding modes (num_modes = 20) for each protein, within a maximum energy range of 5 kcal/mol. The resulting poses were ranked according to their predicted binding affinities (kcal/mol), and the lowest-energy conformation for each ligand–protein complex was selected for further analysis. Visualization of docking poses and generation of two-dimensional and three-dimensional interaction diagrams were performed using Discovery Studio Visualizer v25.1.0.24284 and PyMOL v3.1.6.1 [34], respectively.

### Statistical analysis

All analyses were performed in GraphPad Prism 8.0 for Windows. Normality was assessed with the Shapiro-Wilk test. Datasets meeting the normality assumption were compared by one-way ANOVA followed by the appropriate post-hoc test, whereas datasets not meeting it were compared by the Kruskal-Wallis test followed by Dunn’s post-hoc test. The test applied to each experiment is indicated in the corresponding figure legend. CC_50_ and EC_50_ were calculated by non-linear regression using the log(inhibitor) vs. normalized response – variable slope model, with the Top parameter constrained to 100% and, for the viral replication curves, the Bottom parameter constrained to 0%, consistent with the normalization of all data to the untreated (0.1% DMSO) control [35]. Where 50% cytotoxicity was not reached within the concentration range tested, the CC_50_ is reported as greater than the highest concentration assayed and the corresponding SI as a lower-bound estimate. Differences between groups were considered statistically significant at *p* < 0.05.

## Results

### Pyridine-3-carbohydrazide (P-3-C) is a potent inhibitor of MAYV replication

**Fig. 2:**
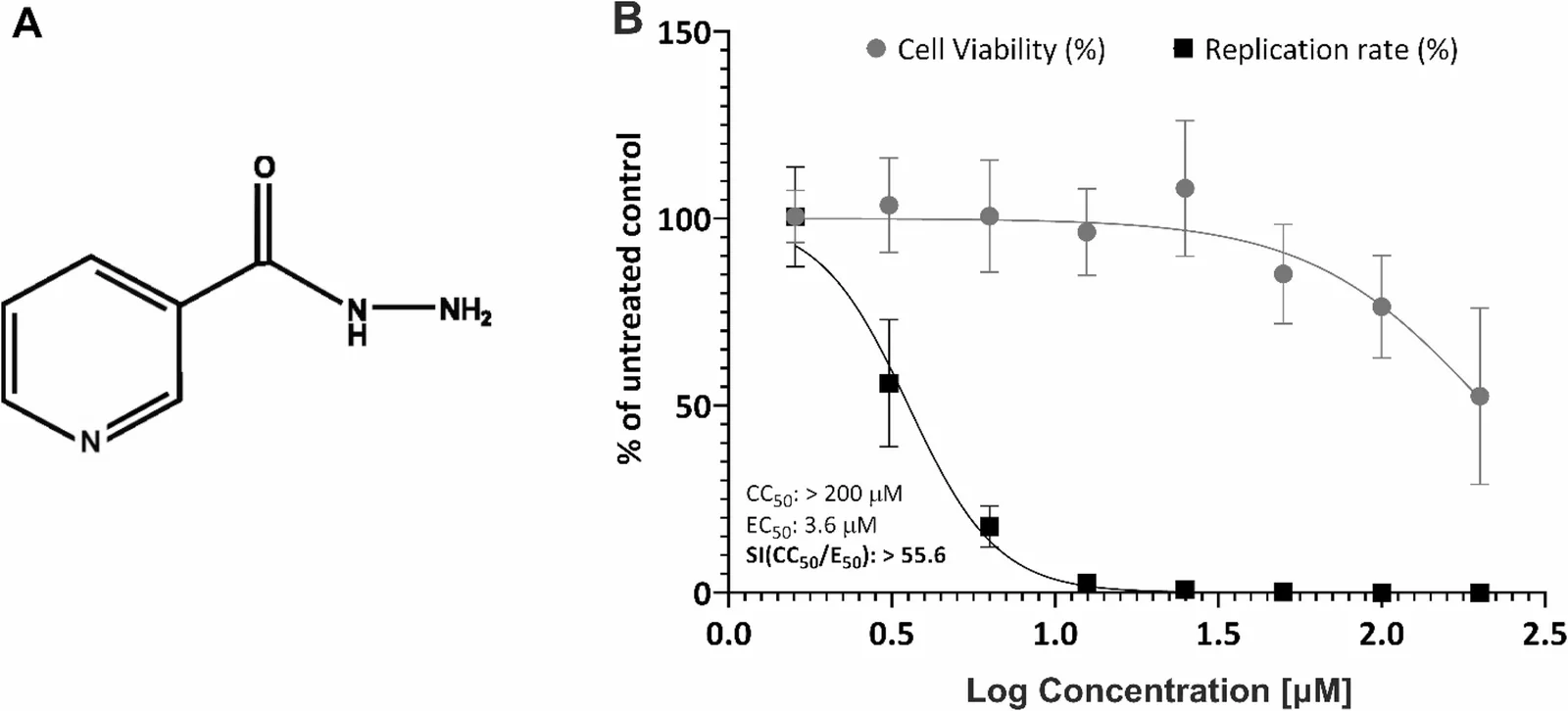
Anti-MAYV activity of P-3-C. **A** Chemical structure of Pyridine-3-carbohydrazide (P-3-C). **B** Vero E6 were infected with MAYV-nLuc-BeAr (MOI 0.1) and treated with P-3-C at concentrations ranging from 1.56 to 200 µM. Viral replication was measured at 24 h.p.i. by nLuc activity (black dots), while cell viability was assessed via WST-I assay (grey dots). The Y-axis shows both readouts as a percentage of the untreated (0.1% DMSO) control processed in parallel, which was set to 100%: viral replication corresponds to nLuc activity in treated cells relative to that control, and cell viability to WST-1 metabolic activity relative to that control. The X-axis shows the log₁₀ of the P-3-C concentration (µM). Data represents the mean ± standard deviation (SD) of three independent experiments (*n* = 3), each performed in triplicate. CC_50_ and EC_50_ values were calculated using non-linear regression in GraphPad Prism 8

To assess the antiviral activity of P-3-C (Fig. 2A), the EC_50_ and CC_50_ of the compound were determined through a dose-response assay. Vero E6 cells were infected with MAYV-nLuc-BeAr at an MOI of 0.1 and simultaneously treated with P-3-C in a two-fold serial dilution ranging from 1.56 to 200 µM. Viral replication was measured at 24 h.p.i., and cell viability was assessed in parallel. The results of these assays demonstrated that P-3-C exhibited an EC_50_ of 3.6 µM, a CC_50_ of > 200 µM (the highest concentration tested; 50% cytotoxicity was not reached), and an SI of > 55.6 (Fig. 2B).

### P-3-C strongly inhibits post-entry stages of MAYV replication

**Fig. 3:**
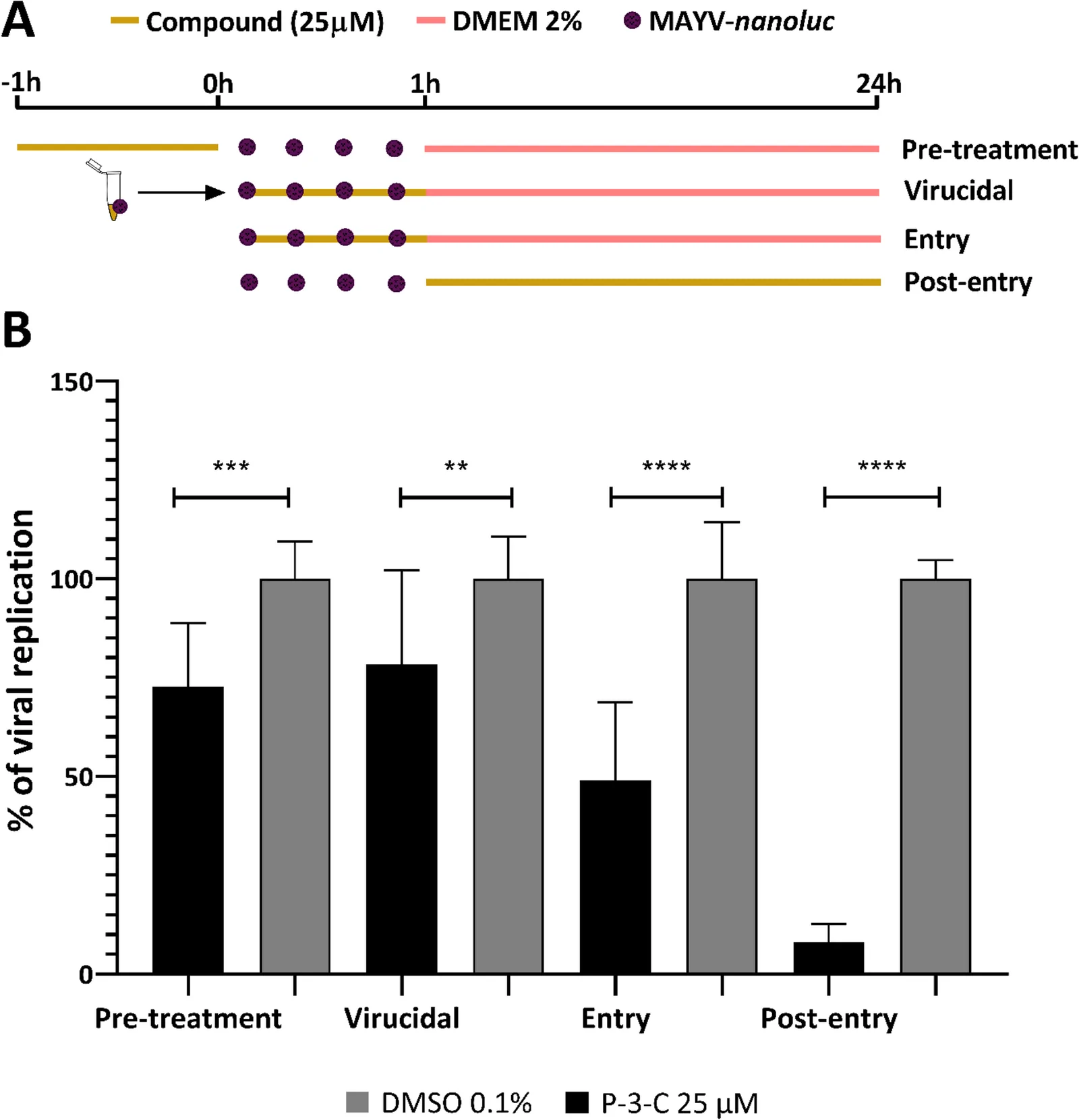
Activity of P-3-C on different stages of MAYV-nLuc BeAr replicative cycle. **A** Schematic representation of the time-of-addition experiments. **B** Pre-treatment: Vero E6 cells were treated with P-3-C for 1 h, infected with MAYV-nLuc-BeAr at MOI 1, and incubated in fresh medium. Virucidal: P-3-C and MAYV-nLuc (MOI 5) were incubated together for 1 h. The inoculum was added to the Vero E6 cells for 1 h, and then the cells were incubated in fresh medium. Entry: Vero E6 cells were infected with MAYV-nLuc-BeAr at MOI 1 and simultaneously treated with P-3-C for 1 h, washed with PBS, and fresh medium was added. Post-entry: Vero E6 cells were infected with MAYV-nLuc-BeAr at MOI 1 for 1 h, washed with PBS, and treated with P-3-C. All infections were quantified at 24 h.p.i by measurement of luminescence levels. Data represent mean ± SD of three independent experiments (*n* = 3), each performed in triplicate, **, *P* < 0.01; ***, *P* < 0.001; ****, *P* < 0.0001 (one-way ANOVA)

Time-of-drug-addition assays **(**Fig. 3A**)** were performed to investigate the effects of P-3-C on different stages of MAYV replication. In all assays, Vero E6 cells were infected with MAYV-nLuc BeAr, treated with P-3-C at 25 µM, and virus replication rates were evaluated at 24 h.p.i. P-3-C demonstrated notable antiviral activity across multiple stages of the MAYV-*nanoluc* replication cycle. Pre-treatment of Vero E6 cells with P-3-C led to a 27% reduction in viral replication (*p* = 0.0006), indicating a mild protective effect. In the virucidal protocol, P-3-C achieved a 22% decrease in MAYV replication (*p* = 0.008). For the viral entry stage, simultaneous administration of P-3-C during infection resulted in a 51% reduction in viral replication (*p* < 0.0001), suggesting moderate inhibition of viral entry. The most pronounced effect was observed in the post-entry phase, where P-3-C produced a substantial 92% decrease in viral replication (*p* < 0.0001), highlighting its greatest efficacy at this stage of the replicative cycle (Fig. 3B).

### P-3-C exhibits a considerable effect on the release of MAYV virions

**Fig. 4:**
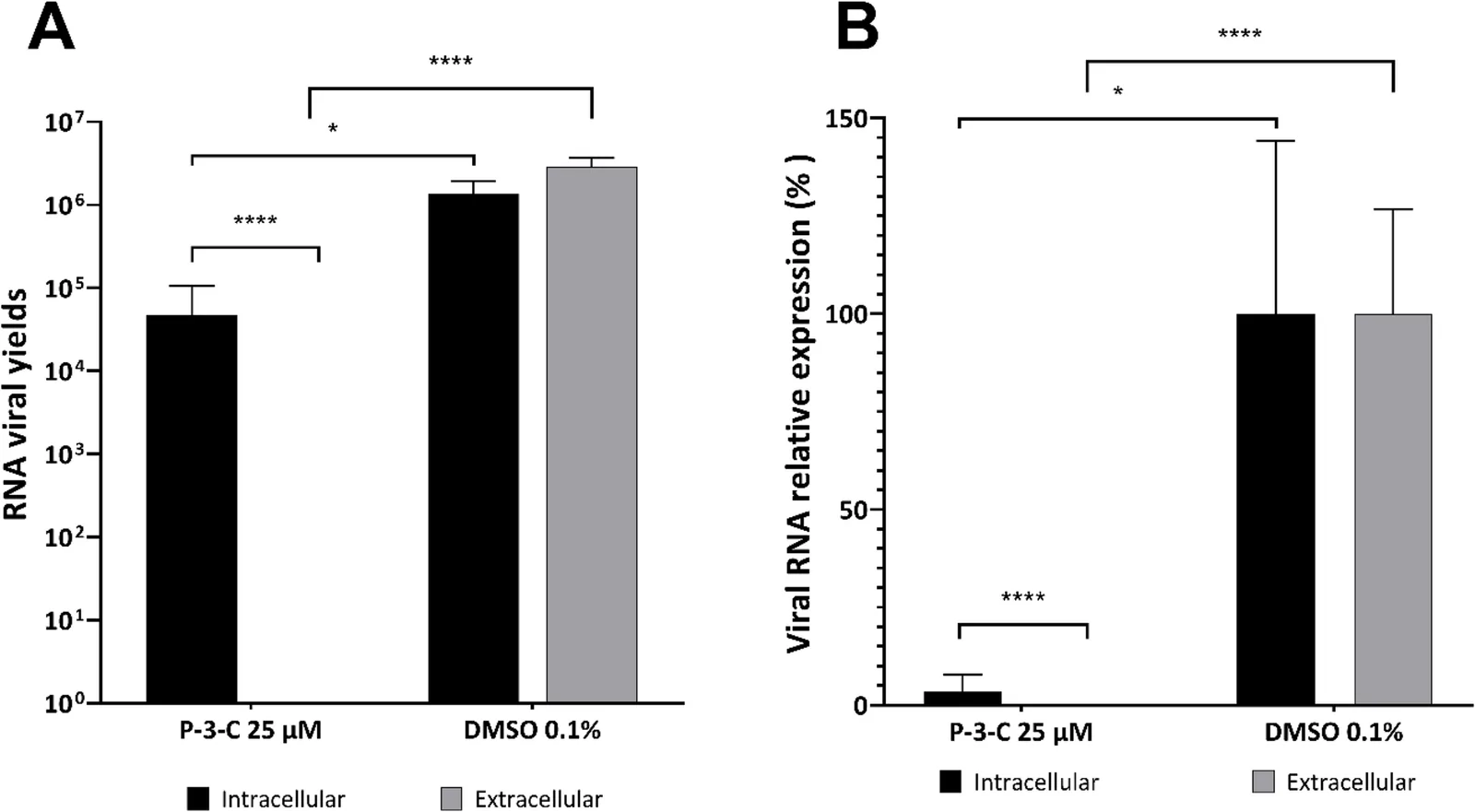
Effect of P-3-C on the release of MAYV virions. Vero E6 cells were infected with MAYV-nLuc-BeAr at an MOI of 1 for 1 h. Washed with PBS and treated with P-3-C at 25 µM or with 0.1% DMSO. At 24 h.p.i., supernatants and cells were collected for RNA extraction. Quantification of viral RNA levels was performed by RT-qPCR. **A** Absolute RNA viral yields and (**B**) Relative viral RNA levels compared to DMSO-treated cells. Data represent the mean ± SD of three independent experiments (*n* = 3) *, *P* < 0.05; ****, *P* < 0.0001 (one-way ANOVA)

To better understand the mechanism of action of P-3-C during the post-entry stages of MAYV infection, Vero E6 cells were infected following the same protocol used in the post-entry assay. However, here the intra-and extracellular viral RNA levels were quantified by RT-qPCR at 24 h.p.i. The results demonstrated that P-3-C significantly reduced viral RNA copy numbers compared to the untreated control (Fig. 4). P-3-C reduced intracellular RNA viral copy numbers by almost 2log_10_ (P-3-C: 4.7 × 10^4^; DMSO: 1.3 × 10^6^; *p* = 0.019) (Fig. 4A), representing 97% inhibition (Fig. 4B). An even more substantial reduction was observed in extracellular RNA levels, resulting in undetectable levels in the supernatant following P-3-C treatment (P-3-C: below detection limit; DMSO: 2.9 × 10^6^; *p* = 0.0001) (Fig. 4A). Thus, the impact of P-3-C on MAYV release from host cells was especially striking, indicating a potent inhibitory effect, with a reduction of extracellular viral RNA to below the limit of detection (Fig. 4).

### P-3-C is effective against different strains of MAYV and CHIKV

Given the significant inhibitory effect of P-3-C on MAYV-nLuc-BeAr replication in Vero cells, we extended our analysis to include a different strain of the virus (MAYV-nLuc-TRVL), a different alphavirus (CHIKV-nLuc-LR), and evaluated the compound’s activity in U-2OS, a human osteosarcoma-derived cell line.

**Fig. 5:**
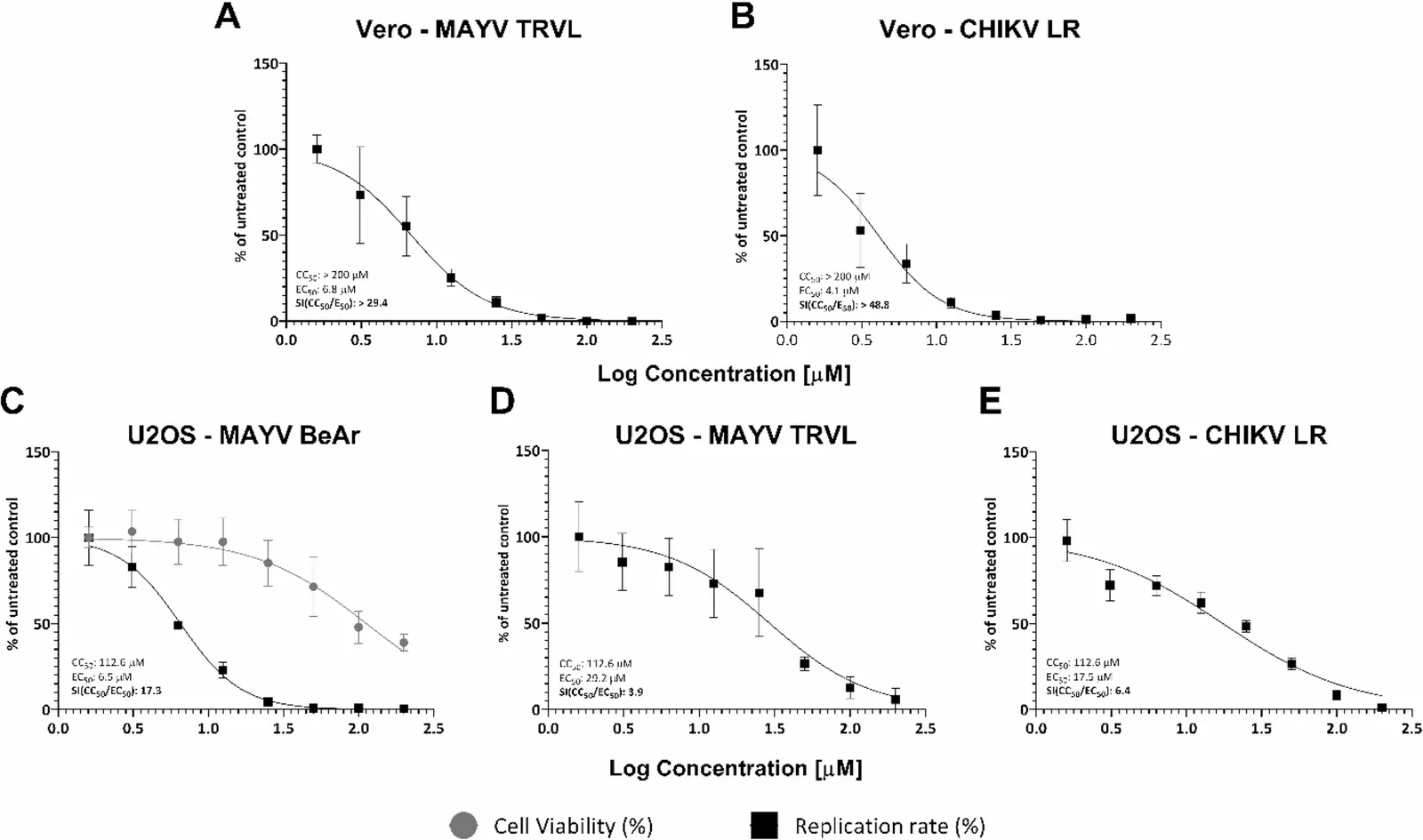
Effects of P-3-C on other different MAYV strains and CHIKV. **A**, **B** Vero E6 cells were infected with MAYV-nLuc-TRVL (A) or CHIKV-nLuc-LR (**B**) at an MOI of 0.1 and treated with P-3-C at concentrations ranging from 1.56 to 200 µM. **C**-**E** U-2OS cells were infected with MAYV-nLuc BeAr (**C**), MAYV-nLuc*-*TRVL (**D**), or CHIKV-nLuc-LR (**E**) at an MOI of 0.1 and treated with P-3-C at the same concentration range. 24 h.p.i. Cells were lysed and analyzed as described for Fig. 2. Cell viability was assessed in parallel using the WST-1 assay (shown on panel C in gray). The Y-axis shows both readouts as a percentage of the untreated (0.1% DMSO) control processed in parallel, which was set to 100%: viral replication corresponds to nLuc activity in treated cells relative to that control, and cell viability to WST-1 metabolic activity relative to that control. The X-axis shows the log₁₀ of the P-3-C concentration (µM). Data represent mean ± SD from two independent experiments (*n* = 2), each performed in quadruplicate. All graphs were generated using GraphPad Prism 8

In Vero E6 cells, P-3-C exhibited EC_50_ of 6.8 µM and 4.1 µM for MAYV-nLuc-TRVL (Fig. 5A) and CHIKV-nLuc-LR (Fig. 5B), resulting in SI of > 29.4 and > 48.8, respectively (SI was calculated based on the CC_50_ results for Vero E6 cells, which exceeded the highest concentration tested (> 200 µM); these SI values are therefore conservative lower-bound estimates). In U-2OS cells, P-3-C showed a CC_50_ of 112.6 µM. For MAYV-nLuc-BeAr, it showed an EC_50_ of 6.5 µM and SI of 17.3 (Fig. 5C); for MAYV-nLuc-TRVL an EC_50_ of 29.2 µM and SI of 3.9 (Fig. 5D), and CHIKV-nLuc-LR, an EC_50_ of 17.5 µM and SI of 6.4 (Fig. 5E). These findings support the effectiveness of P-3-C not only against MAYV BeAr but also against MAYV TRVL strain and CHIKV, suggesting broad activity against different alphavirus strains. However, its efficacy might be cell line-dependent, with higher CC_50_, and consequently SI, in Vero E6 cells than in U-2OS cells.

### P-3-C shows replicon inhibition and binding affinity, suggesting interaction with non-structural proteins

**Fig. 6:**
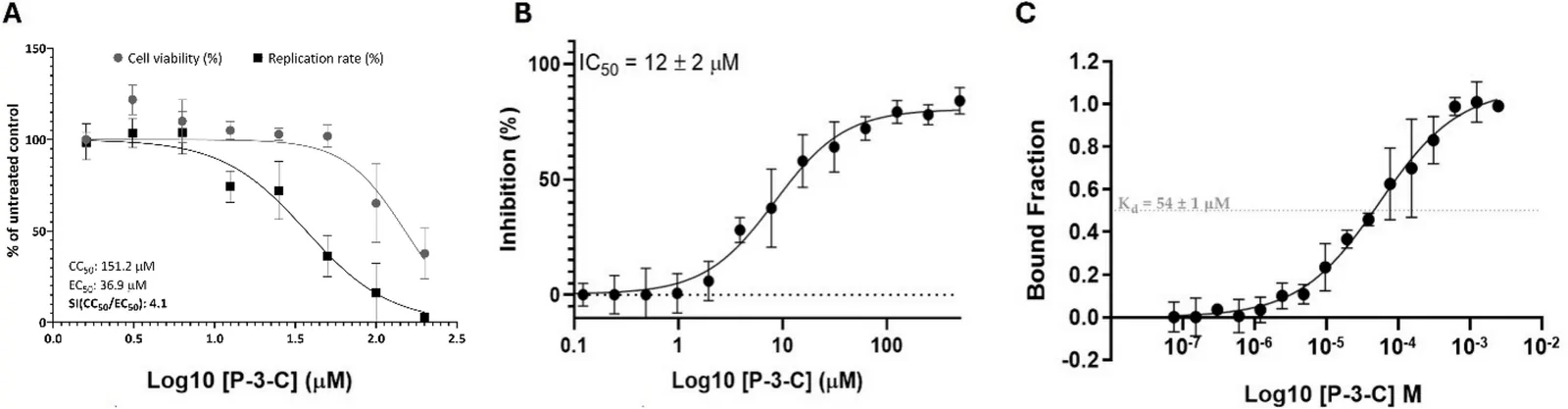
P-3-C suppresses non-structural CHIKV proteins. **A** U-2OS-CHIVsg cells were incubated with P-3-C for 48 h at concentrations ranging from 1.56 to 200 µM. Cells were lysed using Passive Lysis Buffer, and nLuc activity (black) was measured. Cell viability was assessed in parallel using the WST-1 assay (gray). The Y-axis shows replicon-driven nLuc activity and cell viability (WST-1) as a percentage of the untreated (0.1% DMSO) control processed in parallel, and the X-axis shows the log_10_ of the P-3-C concentration (µM). Data represents the mean ± SD of three independent experiments (*n* = 3), each performed in triplicate. Graph generated using GraphPad Prism 8. **B** Concentration-response curve for the inhibition of nsP2pro activity by compound P-3-C. The graph illustrates the percentage of enzymatic inhibition as a function of the logarithmic concentration of compound P-3-C (µM). Data represent the mean ± SD of three independent replicates. The solid line indicates the non-linear regression fit (GraphPad Prism 8.4.3). **C** Concentration-response curve for the binding affinity evaluation of nsP4 for compound P-3-C. The graph illustrates the bound fraction as a function of the logarithmic concentration of compound P-3-C (µM). Data represent the mean ± SD of two independent experiments. The solid line indicates the non-linear regression fit (GraphPad Prism 8.4.3)

Given that P-3-C demonstrated post-entry activity and significant inhibition of CHIKV, its potential effects on CHIKV replication were investigated using a stable U-2OS-CHIKVsg cell line harboring a CHIKV-Repl-nLuc subgenomic replicon, which expresses only the nonstructural proteins of this virus. The analysis revealed a CC_50_ of 151.2 µM and an EC_50_ of 36.9 µM, resulting in a selectivity index (SI) of 4.1 (Fig. 6A).

To further explore this activity on the nonstructural proteins, CHIKV nsP2 (protease) and nsP4 (polymerase) were cloned and heterologous expressed into *E. coli* and Sf9 cells, respectively [29, 32]. Proteins purifications were performed using an AKTA Purifier System (GE Healthcare) (Fig. S3 and S4), and their concentration was quantified by spectrophotometry. The determined IC_50_ of P-3-C against CHIKV nsP2 was 12 µM ± 2 (Fig. 6B), which agrees with the potency observed in the replicon inhibition assay. Furthermore, the assessed binding affinity between nsP4 and P-3-C indicated a *K*_d_ value of 54 µM ± 1 (Fig. 6C). Collectively, these assays indicated that P-3-C shows inhibitory activity of nsP2 and binding affinity for nsP4, thereby suggesting that both the viral protease and polymerase are potential molecular targets for P-3-C.

### In silico interactions of P-3-C with CHIKV and MAYV nsP2 and nsP4

To investigate the molecular interactions between P-3-C and nsP2 and nsP4 of CHIKV and MAYV, molecular docking simulations were performed.

Pairwise global alignment of the sequences used in this study showed that CHIKV and MAYV share 70.7% amino-acid identity (83.4% similarity) in nsP2 and 79.5% identity (86.9% similarity) in nsP4 (Supplementary Table S1; Fig. S1). The validation metrics for the best three-dimensional models (pLDDT, pTM, and PAE) generated by AlphaFold3 for the nsP2 and nsP4 protein structures of both viruses are available in the Supplementary Material (Fig. S2), demonstrating that the structures used for the docking analyses exhibited parameters consistent with high-confidence structural models.

**Fig. 7:**
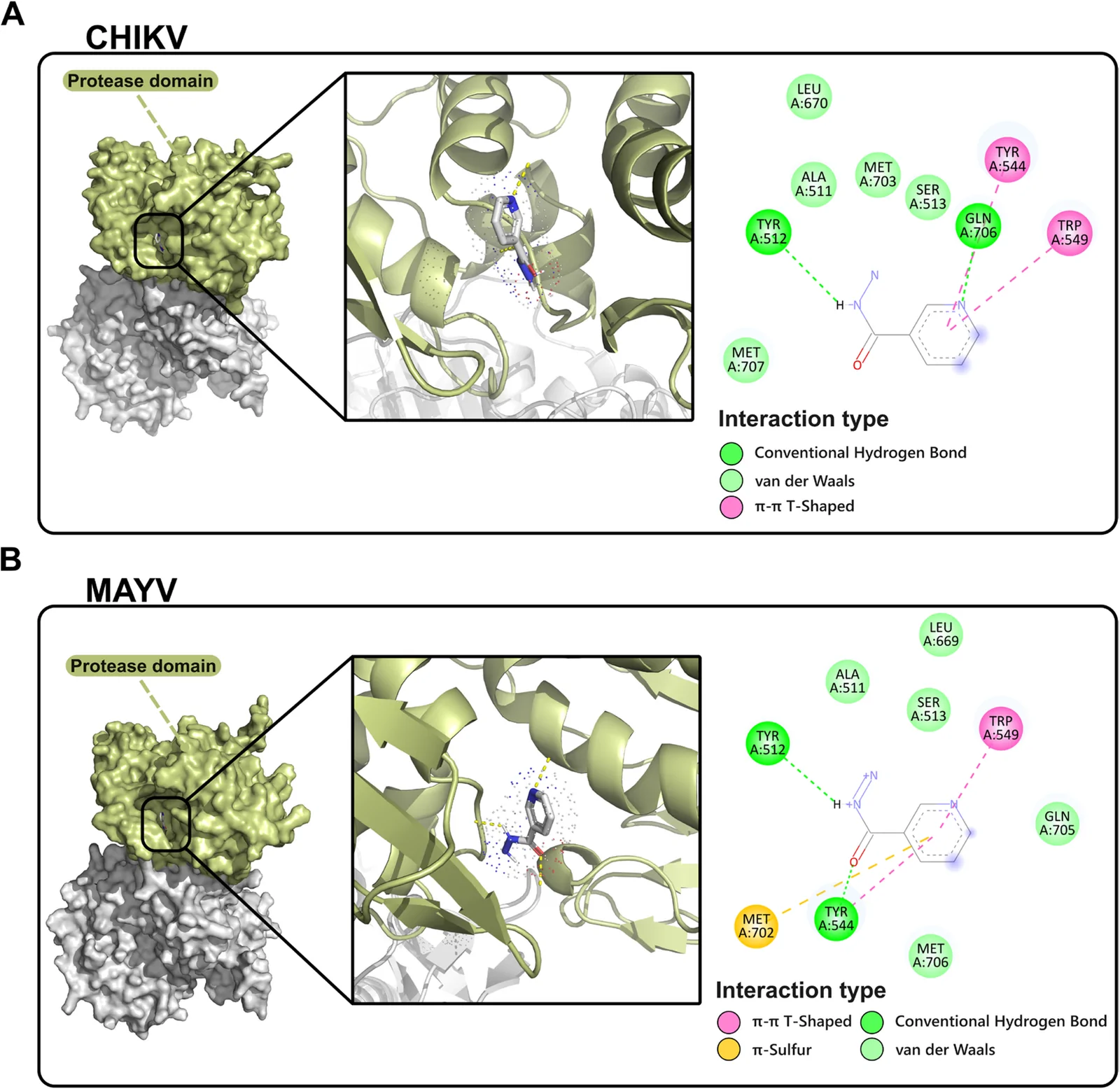
Molecular docking of the compound with the nsP2 protease of CHIKV and MAYV. **A** Docking pose of the compound within the protease domain of the CHIKV nsP2 protein. The left panel shows the overall structure of nsP2, highlighting the protease domain (green) and the ligand-binding pocket. The center panel presents a three-dimensional close-up of the predicted binding mode within the active-site cavity. The right panel illustrates the corresponding two-dimensional interaction map, including conventional hydrogen bonds, π-π T-shaped interactions, and van der Waals contacts between the ligand and surrounding amino acid residues. **B** Docking pose of the compound within the protease domain of the MAYV nsP2 protein. The left panel displays the overall protein structure with the protease domain highlighted in green and the binding pocket indicated. The center panel shows a close-up view of the docked ligand in the predicted binding cavity. The right panel depicts the two-dimensional interaction profile, highlighting conventional hydrogen bonds, π-π T-shaped interactions, π-sulfur interactions, and van der Waals contacts contributing to ligand stabilization

Simulations indicated that the compound was accommodated within the protease domain of nsP2 from both CHIKV and MAYV, occupying a binding pocket located near functionally relevant residues of the catalytic site (Fig. 7A–B). For CHIKV nsP2, the ligand established two conventional hydrogen bonds with Tyr512 and Gln706, in addition to π-π T-shaped interactions with Tyr544 and Trp549. Complex stabilization was further supported by multiple van der Waals interactions involving Ala511, Ser513, Leu670, Met703, and Met707, suggesting a stable binding mode within the catalytic pocket. Similarly, in MAYV nsP2, the ligand occupied the same structural region, forming one conventional hydrogen bond with Tyr512 and one π-π T-shaped interaction with Trp549. Additionally, a π-sulfur interaction with Met702 and a second aromatic interaction and conventional hydrogen bond with Tyr544 contributed to ligand anchoring. The complex was further stabilized by van der Waals interactions with Ala511, Ser513, Leu669, Gln705, and Met706. Overall, the interaction profiles observed for CHIKV and MAYV reveal a conserved binding mode within the viral protease, with only minor differences involving residues surrounding the binding cavity. The best-scoring poses showed predicted binding affinities of − 5.1 kcal/mol for CHIKV and − 5.0 kcal/mol for MAYV (Supplementary Table S2).

**Fig. 8:**
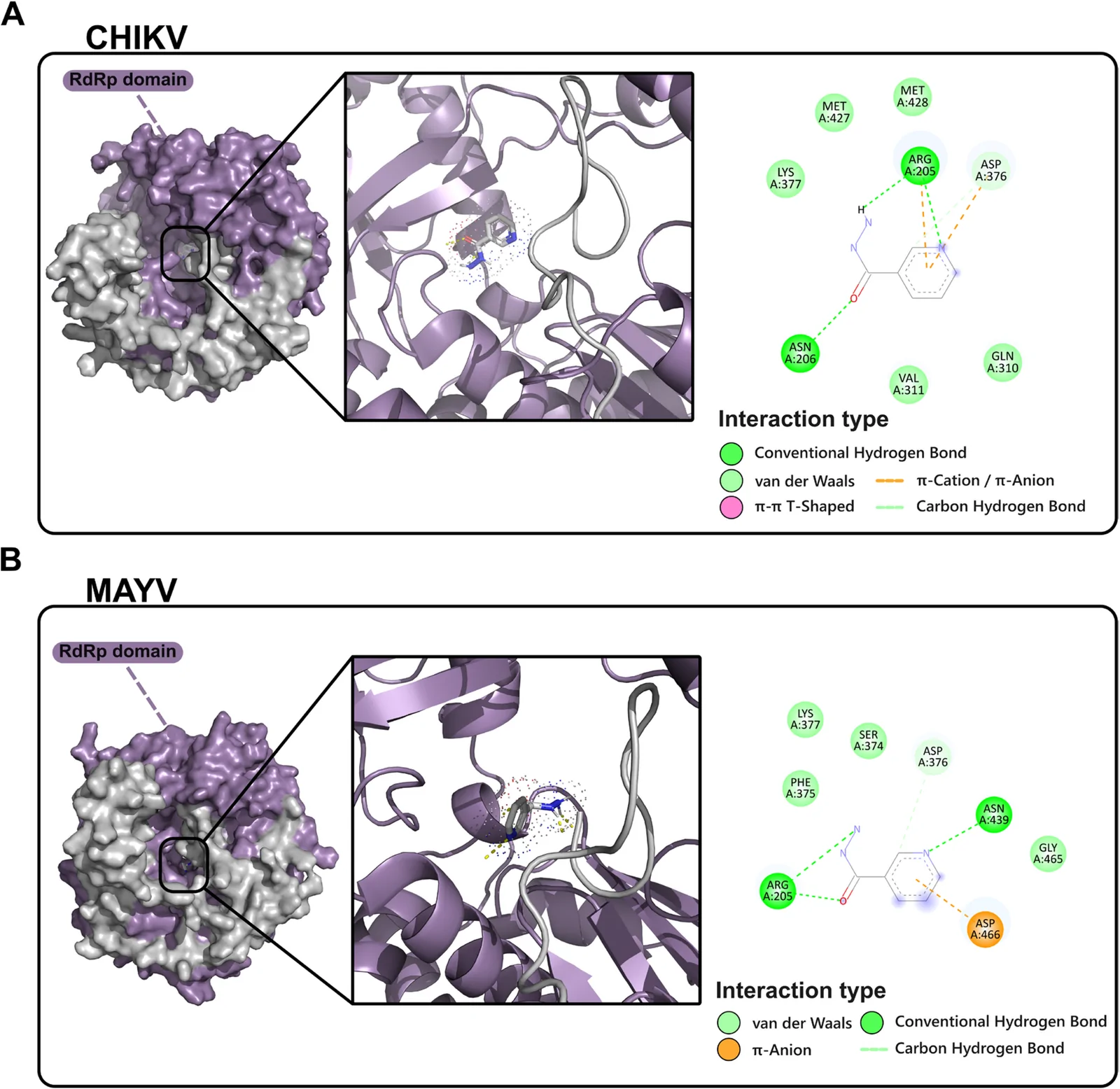
Molecular docking of the compound with the RNA-dependent RNA polymerase (nsP4) of CHIKV and MAYV. **A** Docking pose of the compound within the RdRp domain of CHIKV nsP4. The left panel presents the overall structure of nsP4, with the RNA-dependent RNA polymerase (RdRp) domain highlighted in purple and the predicted binding pocket indicated. The center panel provides a three-dimensional view of the docked ligand within the binding cavity. The right panel shows the two-dimensional interaction map, including conventional hydrogen bonds, carbon hydrogen bonds, π-cation/π-anion interactions, and van der Waals contacts. **B** Docking pose of the compound within the RdRp domain of MAYV nsP4. The left panel shows the overall structure of nsP4 with the RdRp domain highlighted in purple and the ligand-binding site indicated. The center panel displays the predicted binding mode of the ligand within the polymerase cavity. The right panel illustrates the two-dimensional interaction network, highlighting conventional hydrogen bonds, carbon hydrogen bonds, π-anion interactions, and van der Waals contacts involved in ligand stabilization

Docking analyses of nsP4 showed that the P-3-C binds within a cavity located in the RdRp domain of both viruses, forming an interaction network predominantly mediated by hydrogen bonds and electrostatic contacts (Fig. 8A–B). For CHIKV nsP4, the ligand formed conventional hydrogen bonds with Arg205 and Asn206, as well as a carbon hydrogen bond with Asp376. In addition, a π-cation/π-anion interaction involving Arg205 and Asp376 was identified, suggesting an additional contribution to complex stabilization. Neighboring residues, including Lys377, Met427, Met428, Val311, and Gln310, further stabilized the complex through van der Waals interactions. For MAYV nsP4, the ligand exhibited a similar interaction pattern, establishing conventional hydrogen bonds with Arg205 and Asn439, together with a carbon hydrogen bond involving Asp376. A π-anion interaction with Asp466 was detected. Additional van der Waals interactions with Phe375, Lys377, Ser374, and Gly465 also contributed to complex stabilization. The best-scoring poses showed predicted binding affinities of − 5.3 kcal/mol for CHIKV and − 4.9 kcal/mol for MAYV (Supplementary Table S2).

Taken together, docking results indicate that the compound exhibits a binding mode toward both the nsP2 protease and the nsP4 RdRp of CHIKV and MAYV. Although subtle differences were observed in peripheral residues participating in ligand recognition, the key molecular interactions were largely preserved between the two viruses. These findings suggest that the compound targets structurally conserved regions of both viral proteins.

## Discussion

MAYV is an arbovirus of increasing public health concern due to its capacity to cause outbreaks across the Americas [36]. Cases have been detected in more than 11 countries, with Brazil, Peru, and Venezuela reporting the highest numbers [36]. Diagnosis remains challenging because the clinical symptoms of MAYV closely resemble those of dengue and CHIKV. Currently, no approved antiviral treatments exist to manage the debilitating symptoms and severe chronic complications associated with MAYV, such as arthralgia and hemorrhagic manifestations [37]. Therefore, the development of effective antiviral therapies targeting MAYV is crucial [37]. In this context, the discovery of the antiviral effects of P-3-C against MAYV, particularly its prominent impact on the post-entry stages such as viral RNA replication and virion production, along with its modest selectivity index, represents an important advancement.

Pyridine and hydrazides are organic compounds of great importance in medicinal chemistry [38]. Pyridine, a nitrogenous heterocycle, serves as the backbone for more than 50 FDA-approved drugs, including treatments for inflammation and cancer, and is known for its antioxidant properties [38]. Hydrazides, characterized by their versatile functional groups (NH₂NHC = O, NHNH₂, and NHN = CH-), provide a flexible platform for the design and synthesis of bioactive compounds [19]. Within this family, P-3-C has been mainly used as a structural core for synthesis, though it also exhibits antimicrobial properties, inhibiting the synthesis of mycolic acids, essential components of the bacterial cell wall [19]. However, its positional isomer, P-4-C (isoniazid), is widely recognized as a clinical agent. Additionally, diverse derivates from these hydrazide scaffolds have shown significant antibacterial and antifungal activities, which are attributed to the hydrazide moiety in their structures [16–18].

Despite these diverse biological effects, P-3-C has not been previously evaluated as an antiviral agent, highlighting the importance of this study. A previous study utilized its isomer, isoniazid, as a ligand to develop two copper complexes (Cu+-INH and Cu++-INH), which exhibited notable inhibition of Rous Sarcoma Virus (RSV) replication [39]. However, RSV is a retrovirus whose replication relies on reverse transcription and genome integration, mechanisms fundamentally distinct from those of alphaviruses. Therefore, these findings indicate that hydrazide-containing scaffolds can be incorporated into antiviral chemical frameworks, supporting the rationale for investigating P-3-C as a potential antiviral compound.

In our study, P-3-C demonstrates a strong activity in MAYV post-entry stages, including a remarkable decrease in copy numbers in both intra- and extracellular RNA levels, suggesting a strong effect on MAYV RNA replication and budding of host cells. Similar properties were seen for Fac4, a natural acridone compound that inhibited the release of hepatitis C virus from Huh 7.5 cells [40]. Antiviral assays conducted using a U-2OS-CHIKVsg replicon suggested that P-3-C may be targeting the RNA replication of alphaviruses. Although the overall identity between both proteins is moderate, conservation is essentially complete at the sites relevant to the present work: the nsP2 catalytic residues Cys478, Trp479 and His548 are strictly conserved between the two viruses, as is the GDD catalytic motif of the nsP4 RdRp, and all nine residues forming the P-3-C binding pocket in CHIKV nsP2 (Ala511, Tyr512, Ser513, Tyr544, Trp549, Leu670, Met703, Gln706 and Met707) are identical in MAYV. The single difference within the catalytic region, Ser482 in CHIKV corresponding to Cys in MAYV, occurs at a position previously shown not to be required for nsP2 protease activity. Given this conservation of the catalytic and ligand-binding sites [30], we further investigated whether P-3-C interacts with these viral non-structural proteins, using the CHIKV enzymes as a surrogate for, rather than the equivalent of, their MAYV counterparts. To this end, MST and enzymatic inhibition assays were performed with recombinant CHIKV nsP4 and nsP2, respectively, in the presence of P-3-C. The MST assay confirmed that P-3-C binds to CHIKV nsP4 with low-micromolar affinity. In addition, P-3-C potently inhibited the protease activity of CHIKV nsP2, a cysteine protease essential for viral polyprotein processing [41]. Together, these findings indicate that P-3-C inhibits nsP2 activity and binds nsP4 at concentrations comparable to those observed in the replicon assay. This supports a potential multitarget mechanism of action.

A previous in silico study performed by our group found that P-3-C, when combined with another molecule, exhibited greater stability and binding affinity to the nsP9 protein of SARS-CoV-2 [42]. Our molecular docking studies revealed the potential interactions between P-3-C and both nsP2 and nsP4 of MAYV and CHIKV. nsP2 is a multifunctional enzyme with an N-terminal helicase/NTPase/RNA triphosphatase domain and a C-terminal papain-like cysteine protease domain [43]. The protease is responsible for the sequential processing of the nsP1234 polyprotein, and its activity is therefore indispensable for the maturation of the viral replication complex; substitution of the catalytic residue Cys478 abolishes polyprotein processing, RNA replication and the recovery of infectious virus [41, 44]. In our docking simulations, P-3-C was accommodated within the catalytic cleft of nsP2, establishing hydrogen bonds and hydrophobic contacts with residues surrounding the catalytic site, and this prediction was supported experimentally by the inhibition of CHIKV nsP2 protease activity in the FRET-based assay. Notably, P-3-C inhibited nsP2 at a concentration lower than that required for nsP4 binding, suggesting that the protease may be the more sensitive of the two targets. Because inhibition of the nsP2 protease would prevent the correct processing of the replicase precursor, it would compromise viral replication at a step upstream of RNA synthesis itself. nsP4, in turn, possesses an N-terminal region important for protein-protein interactions and template recognition [45]. Also, it has a C-terminal region containing the RdRp domain, which constitutes the polymerase’s catalytic core [46]. Its functions include the synthesis of genomic and subgenomic RNA, maintenance, and repair of the poly A tail [47]. Therefore, inhibition of this region by a compound would disrupt the proper functioning of the replication complex, consequently interrupting viral protein synthesis and viral multiplication [48]. Taken together, the engagement of two distinct enzymes acting at consecutive steps of the replication cycle (polyprotein maturation and RNA synthesis) is consistent with the multitarget mechanism proposed here and may explain the strong post-entry effect observed for P-3-C. We emphasize, however, that docking indicates geometric and chemical complementarity rather than proven enzymatic inhibition, and that residue-level validation by site-directed mutagenesis remains necessary.

Additionally, the use of two cell lines, Vero E6 and U-2OS, provides relevant information on the effects of P-3-C. Vero E6 cells, frequently used in virological studies due to their high susceptibility to infection, allowed us to assess the efficacy of the compound in a commonly employed mammalian system [49]. In contrast, U-2OS cells, derived from human osteosarcoma, were used as a permissive human cell model to evaluate alphavirus infection in a human-derived cellular environment. The significant inhibition of both MAYV strains (BeAr and TRVL) and CHIKV (La Reunión strain) highlights P-3-C potential as an antiviral agent. However, an antiviral efficacy cell line-dependent could not be discarded. While P-3-C exhibited robust selectivity in Vero E6 cells, the selectivity indices obtained in U-2OS cells indicate more limited selectivity in this cellular model, warranting further investigation. Furthermore, it is important to acknowledge as a limitation that the antiviral assays in U-2OS cells were performed with only two independent experiments. While these data suggest a clear trend toward decrease replication across different alphavirus strains by P-3-C, further studies should better elucidate the effect of this compound on alphavirus RNA and nsPs.

Here, we also observed a low level, but still significant, protective effect of P-3-C for the cells when the pre-treatment or entry protocols were used. Most likely, these effects can be attributed to potent post-entry effects of the compound absorbed by treated cells. It is well known that alphavirus infection causes a significant increase in intracellular Reactive Oxygen Species (ROS) levels during viral replication, causing oxidative stress in cells and leading to an imbalance in cellular homeostasis [50, 51]. Camini and coworkers found a significant increase in ROS in HepG2 and J774 cells infected with MAYV 6 h.p.i [52]. Likewise, another study carried out in RAW 264.7 cells demonstrated a considerable increase in ROS also 6 h.p.i, which coincided with the peak of viral replication [53]. Although increased ROS is crucial for activating the immune response, an exacerbated increase during viral replication causes an imbalance in cellular redox homeostasis, resulting in loss of cellular integrity and functionality [54]. In this sense, as P-3-C is known for its antioxidant properties [15, 38], it could be reducing ROS levels, improving cell survival, and thus enhancing the antiviral activity against MAYV, as observed by Camini and colleagues, where an antioxidant natural compound could reduce the replication of MAYV and restore the pro-oxidant/antioxidant balance in infected cells [55]. A partial antiviral effect caused by the treatment with P-3-C could be associated with the antioxidant property of this compound. However, this hypothesis was not tested and remains to be elucidated.

In summary, our results demonstrate that P-3-C exhibits antiviral potential against multiple strains of MAYV, being particularly effective during the post-entry stages of the replicative cycle. Additionally, P-3-C demonstrated effectively inhibiting CHIKV replication. Such action against different alphaviruses underscores its potential as a versatile antiviral candidate. While the precise mechanism of action remains a topic for additional validation, these data suggest that both the viral protease and polymerase are potential molecular targets of P-3-C. These findings position P-3-C as a promising agent for further research in the development of anti-MAYV and anti-CHIKV therapies.

## Supplementary Information

Below is the link to the electronic supplementary material.

Supplementary Material 1

## Data Availability

All data supporting the findings of this study are available within the paper.
