# Supplementary material for: Pyridin-3-carbohydrazide strongly impairs Mayaro and Chikungunya virus replication in vitro
Source: Arch Virol. 2026 Aug 12;171(9):252. doi: 10.1007/s00705-026-06709-y (PMC13469200; doi:10.1007/s00705-026-06709-y)
Supplement: Supplementary file 1 [file 705_2026_6709_MOESM1_ESM.docx]

**Supplementary Material**

**Table S1.** Pairwise sequence comparison of the non-structural proteins nsP2 and nsP4 of CHIKV and MAYV.

| **Protein** | **Length (aa)** | **Identity (%)** | **Similarity (%)** | **Catalytic residues** | **Binding-pocket residues** |
| --- | --- | --- | --- | --- | --- |
| nsP2 | 798 | 70.7 | 83.4 | 3/3 conserved | 9/9 conserved |
| nsP4 | 611 | 79.5 | 86.9 | GDD conserved | n.a. |

Sequences were retrieved from UniProt (CHIKV, Q8JUX6, strain S27-African prototype; MAYV, Q8QZ73, strain Brazil) and aligned pairwise using the Needleman–Wunsch global alignment algorithm with the BLOSUM62 substitution matrix. Identity and similarity are expressed as a percentage of the aligned length. Catalytic residues: Cys478, Trp479 and His548 for the nsP2 protease; the GDD motif (residues 465–467) for the nsP4 RdRp. Binding-pocket residues correspond to the nine residues contacting P-3-C in the CHIKV nsP2 docking pose (Ala511, Tyr512, Ser513, Tyr544, Trp549, Leu670, Met703, Gln706 and Met707); all are identical in MAYV. Residue numbering follows the mature CHIKV chains. The single non-conserved position within the catalytic region, Ser482 (Cys in MAYV), is not required for nsP2 protease activity. The complete alignments are shown in **Supplementary Figure S1.**

**Figure S1.** Pairwise sequence alignment of the non-structural proteins nsP2 and nsP4 of chikungunya virus (CHIKV) and Mayaro virus (MAYV). Sequences were retrieved from UniProt (CHIKV, Q8JUX6, strain S27-African prototype; MAYV, Q8QZ73, strain Brazil). As both entries correspond to the complete non-structural polyprotein (P1234), the mature nsP2 (798 residues) and nsP4 (611 residues) chains were extracted according to the chain annotations of each entry. The chains were aligned pairwise using the Needleman-Wunsch global alignment algorithm with the BLOSUM62 substitution matrix. nsP2 (first panel) (70.7% identity, 83.4% similarity). nsP4 (second panel) (79.5% identity, 86.9% similarity). Dark grey shading indicates identical residues and light grey shading indicates conservative substitutions (BLOSUM62 score > 0). Red boxes indicate the catalytic residues: Cys478, Trp479 and His548 of the nsP2 protease, and the GDD motif (residues 465–467) of the nsP4 RNA-dependent RNA polymerase; all of which are strictly conserved between the two viruses. Blue boxes indicate the nine residues that form the P-3-C binding pocket in CHIKV nsP2 (Ala511, Tyr512, Ser513, Tyr544, Trp549, Leu670, Met703, Gln706 and Met707); all are identical in MAYV. Residue numbering follows the mature CHIKV chains. Ser482 of CHIKV, which corresponds to Cys in MAYV, is the only non-conserved position within the catalytic region.

The structural quality assessment of the AlphaFold3-predicted models demonstrated consistently high confidence for both nsP2 and nsP4 proteins of CHIKV and MAYV (**Figure S2**). Among the five models generated for each protein, the top-ranked models were selected for subsequent molecular docking analyses based on their predicted Template Modeling (pTM) scores. The selected nsP2 models exhibited pTM scores of 0.85 for CHIKV and 0.90 for MAYV, whereas the selected nsP4 models achieved pTM scores of 0.94 for both viruses, indicating high overall confidence in the predicted domain organization and global protein architecture. The Predicted Aligned Error (PAE) maps revealed low inter-residue positional uncertainty across most regions of each protein, with increased error primarily restricted to flexible terminal segments and inter-domain regions. Likewise, residue-level confidence (pLDDT) profiles showed that the vast majority of residues presented scores above 90, corresponding to very high local structural confidence, while only a limited number of residues, predominantly located at terminal regions and predicted flexible loops, displayed moderate confidence values. Collectively, these metrics indicate that the selected AlphaFold3 models possess high structural reliability and are suitable for downstream molecular docking analyses.

**Figure S2.** **Structural quality assessment of AlphaFold3-predicted models for CHIKV and MAYV nsP2 and nsP4 proteins.** For each protein, the five AlphaFold3-generated models are ranked according to their predicted Template Modeling (pTM) scores (left panels). The corresponding Predicted Aligned Error (PAE) matrices (center panels) illustrate the expected positional error between residue pairs, with lighter colors indicating lower prediction uncertainty. Residue-level confidence is shown by the predicted Local Distance Difference Test (pLDDT) scores (right panels), where higher values indicate greater confidence in local structural accuracy. Dashed vertical lines denote the predicted domain boundaries separating the N-terminal region from the protease domain in nsP2 and from the RNA-dependent RNA polymerase (RdRp) domain in nsP4. The highest-ranked model for each protein was selected for subsequent molecular docking analyses.

**Figure S3. SDS–PAGE analysis of nsP2pro purification.** MW, molecular-weight marker; lane 1, soluble fraction; lane 2, insoluble fraction; lane 3, flow-through from the first affinity chromatography step (AF1); lane 4, AF1 elution; lane 5, post-dialysis sample; lane 6, flow-through from the second affinity chromatography step (AF2); lane 7, AF2 elution with 10 mM imidazole; lanes 8–13, size-exclusion chromatography fractions. The yellow arrow indicates His-tagged nsP2pro (~50 kDa), and the green arrows indicate cleaved nsP2pro (~37 kDa).

**Figure S4. SDS–PAGE analysis of nsP4 purification. (A**) IMAC: immobilized metal affinity chromatography; HAC: heparin affinity chromatography; MW, molecular weight marker; lane 1, insoluble fraction; lane 2, flow through; lane 3, wash; lane 4, elution; lane 5, inject; lane 6, flow-through; lanes 7 and 8 elution. (**B**) IEC: Ion Exchange Chromatography; lanes 1 and 2 nsp4-chkv elution. Red Arrows indicate the eluted protein fractions

**Table S2.** Predicted binding affinities of pyridin-3-carbohydrazide (P-3-C) for the non-structural proteins nsP2 and nsP4 of CHIKV and MAYV, obtained by molecular docking with AutoDock Vina.

**(A) nsP2**

| **Pose** | **CHIKV nsP2** | | | **MAYV nsP2** | | |
| --- | --- | --- | --- | --- | --- | --- |
|  | **Affinity (kcal/mol)** | **RMSD l.b. (Å)** | **RMSD u.b. (Å)** | **Affinity (kcal/mol)** | **RMSD l.b. (Å)** | **RMSD u.b. (Å)** |
| 1 | -5.1 | 0.000 | 0.000 | -5.0 | 0.000 | 0.000 |
| 2 | -4.6 | 8.803 | 10.387 | -4.7 | 7.304 | 9.634 |
| 3 | -4.6 | 8.677 | 10.367 | -4.7 | 7.959 | 10.237 |
| 4 | -4.5 | 5.894 | 7.411 | -4.4 | 5.882 | 7.477 |
| 5 | -4.3 | 9.086 | 10.722 | -4.4 | 3.981 | 5.072 |
| 6 | -4.3 | 2.974 | 3.999 | -4.4 | 7.135 | 9.464 |
| 7 | -4.2 | 10.204 | 11.226 | -4.4 | 1.518 | 1.658 |
| 8 | -4.2 | 10.559 | 11.432 | -4.3 | 5.021 | 7.394 |
| 9 | -4.2 | 1.813 | 2.195 | -4.3 | 10.398 | 11.290 |
| 10 | -4.2 | 10.133 | 11.137 | -4.3 | 2.937 | 5.295 |
| 11 | -4.2 | 10.645 | 10.875 | -4.2 | 9.456 | 11.155 |
| 12 | -4.1 | 4.298 | 5.455 | -4.2 | 2.064 | 2.651 |
| 13 | -4.0 | 14.248 | 15.071 | -4.2 | 9.158 | 11.457 |
| 14 | -4.0 | 10.541 | 11.376 | -4.0 | 10.001 | 11.046 |
| 15 | -4.0 | 13.483 | 15.063 | -4.0 | 9.833 | 11.756 |
| 16 | -3.8 | 14.475 | 15.103 | -4.0 | 9.444 | 11.142 |
| 17 | -3.8 | 9.194 | 11.049 | -4.0 | 9.597 | 11.233 |
| 18 | -3.7 | 15.128 | 15.914 | -3.9 | 11.305 | 12.434 |
| 19 | -3.7 | 14.929 | 15.747 | -3.9 | 10.584 | 11.476 |
| 20 | -2.4 | 15.784 | 17.573 | -3.9 | 9.661 | 10.529 |

**(B) nsP4**

| **Pose** | **CHIKV nsP4** | | | **MAYV nsP4** | | |
| --- | --- | --- | --- | --- | --- | --- |
|  | **Affinity (kcal/mol)** | **RMSD l.b. (Å)** | **RMSD u.b. (Å)** | **Affinity (kcal/mol)** | **RMSD l.b. (Å)** | **RMSD u.b. (Å)** |
| 1 | -5.3 | 0.000 | 0.000 | -4.9 | 0.000 | 0.000 |
| 2 | -5.1 | 15.584 | 16.196 | -4.7 | 9.408 | 10.047 |
| 3 | -5.1 | 14.047 | 15.242 | -4.6 | 9.747 | 10.722 |
| 4 | -5.0 | 1.279 | 1.695 | -4.5 | 10.248 | 10.816 |
| 5 | -4.8 | 15.258 | 15.711 | -4.5 | 1.765 | 1.972 |
| 6 | -4.8 | 14.176 | 14.693 | -4.5 | 10.064 | 10.524 |
| 7 | -4.8 | 5.361 | 6.279 | -4.4 | 5.653 | 5.997 |
| 8 | -4.7 | 2.774 | 3.740 | -4.3 | 2.221 | 4.217 |
| 9 | -4.7 | 5.149 | 6.293 | -4.3 | 10.079 | 10.549 |
| 10 | -4.6 | 3.461 | 5.020 | -4.3 | 11.443 | 12.188 |
| 11 | -4.4 | 3.630 | 4.251 | -4.3 | 4.639 | 6.093 |
| 12 | -4.4 | 2.332 | 3.863 | -4.2 | 10.858 | 11.331 |
| 13 | -4.3 | 10.375 | 11.335 | -4.2 | 3.420 | 4.604 |
| 14 | -4.3 | 2.163 | 2.932 | -4.2 | 10.679 | 11.221 |
| 15 | -4.2 | 14.338 | 14.824 | -4.0 | 9.455 | 10.071 |
| 16 | -4.2 | 5.302 | 6.841 | -4.0 | 5.511 | 7.021 |
| 17 | -4.1 | 14.549 | 15.051 | -4.0 | 4.047 | 5.267 |
| 18 | -4.0 | 13.178 | 14.801 | -4.0 | 7.134 | 7.393 |
| 19 | -4.0 | 14.032 | 15.420 | -4.0 | 5.185 | 6.154 |
| 20 | -4.0 | 9.091 | 9.871 | -3.9 | 5.003 | 6.512 |

All binding modes generated for each ligand–protein complex are listed, ranked by predicted binding affinity. Docking was performed with AutoDock Vina using an exhaustiveness value of 24, with search boxes centred on the catalytic residues (Cys478 for the nsP2 protease and Asp466, within the GDD motif, for the nsP4 RdRp). RMSD l.b. and RMSD u.b. denote the lower- and upper-bound root-mean-square deviations of each pose relative to the best-scoring pose, which is therefore assigned an RMSD of 0.000 Å. The lowest-energy conformation of each complex (pose 1) was selected for the interaction analyses shown in Figures 7 and 8. Predicted binding affinities are reported in kcal/mol; more negative values indicate a more favourable predicted interaction.
